# The furosemide stress test predicts the timing of continuous renal replacement therapy initiation in critically ill patients with acute kidney injury: a double-blind prospective intervention cohort study

**DOI:** 10.1186/s40001-023-01092-9

**Published:** 2023-04-05

**Authors:** Kun Zhang, Haohua Zhang, Chai Zhao, Zhenjie Hu, Jiuyan Shang, Yuhong Chen, Yan Huo, Congcong Zhao, Bin Li, Suzhi Guo, the Hebei Key Laboratory of Critical Disease Mechanism and Intervention

**Affiliations:** 1 Department of Critical Care Medicine, The Fourth Hospital of Hebei Medical University, Shijiazhuang, Hebei China; 2 Department of Emergency, Xian People’s Hospital, Xian, Shanxi China; 3 Department of Pathology, The Fourth Hospital of Hebei Medical University, Shijiazhuang, Hebei China

**Keywords:** Critical care medicine, Acute kidney injury, Furosemide stress test, Continuous renal replacement therapy

## Abstract

**Background:**

Continuous renal replacement therapy (CRRT) remains a crucial treatment for critically ill patients with acute kidney injury (AKI), although the timing of its initiation is still a matter of contention. Furosemide stress testing (FST) may be a practical and beneficial prediction instrument. This research was meant to examine if FST can be used to identify high-risk patients for CRRT.

**Methods:**

This study is a double-blind, prospective interventional cohort study. For patients with AKI receiving intensive care unit (ICU) income, FST was selected with furosemide 1 mg/kg intravenous (1.5 mg/kg intravenous if a loop diuretic was received within 7 days). Urinary volume more than 200 ml at 2 h after FST was FST-responsive, less than 200 ml was FST-nonresponsive. The FST results are kept strictly confidential from the clinician, who decides whether to initiate CRRT based on laboratory testing and clinical symptoms other than the FST data. The FST data are concealed from both the patients and the clinician.

**Results:**

FST was delivered to 187 of 241 patients who satisfied the inclusion and exclusion criteria, with 48 patients responding to the test and 139 patients not responding. 18/48 (37.5%) of the FST-responsive patients received CRRT, while 124/139 (89.2%) of the FST-nonresponsive patients received CRRT. There was no significant difference between the CRRT and non-CRRT groups in terms of general health and medical history (*P* > 0.05). Urine volume after 2 h of FST was considerably lower in the CRRT group than in the non-CRRT group (35 ml, IQR5-143.75 versus 400 ml, IQR210-890; *P* = 0.000). FST non-responders were 2.379 times more likely to initiate CRRT than FST responders (95% CI 1.644–3.443, *P* = 0.000). The area under the curve (AUC) for initiating CRRT was 0.966 (cutoff of 156 ml, sensitivity of 94.85%, specificity of 98.04%, *P* < 0.001).

**Conclusion:**

This study demonstrated that FST is a safe and practical approach for predicting the initiation of CRRT in critically ill AKI patients.

***Trial registration*** www.chictr.org.cn, ChiCTR1800015734, Registered 17 April 2018.

## Background

Acute kidney injury (AKI) in critically ill patients has long been a major complication for intensive care unit (ICU) physicians due to its morbidity of 57.3% and mortality of 26.9% [1]. Continuity renal replacement therapy (CRRT) is the standard treatment for severe AKI and its usage for life-threatening complications (e.g., severe hyperkalemia), but the results of major randomized studies on the date of CRRT initiation remain unclear [2–5]. The AKIKI trial categorized AKI stage 3 as the early group compared to patients with urgent reasons to commence CRRT and did not discover better mortality in the early group, most likely because it was too late to start CRRT [2]. The ELAIN trial identified phase 2 of AKI as an early group, demonstrating a substantial benefit over commencing CRRT in phase 3. Obviously, the addition of NGAL also provides us with fresh ideas [3]. Since the AKIKI and ELAIN investigations finished abruptly, the STARRT-AKI study included both ICU patients with renal insufficiency and AKI stage 3; nonetheless, no CRRT improved patient outcome [4], and Bayesian secondary analysis did not demonstrate superiority for early beginning of CRRT [5].

As is the case with the design of these massive research, the terms "early" and "delayed" have distinct meanings. The basis on which clinicians determine whether to commence CRRT is also fundamentally diverse, and in the same patient, various physicians may decide differently whether to start CRRT. The reason for this is that, to far, no comparable troponin marker has an extraordinarily high specificity and sensitivity for identifying myocardial infarct. It has been established that furosemide stress testing (FST) may predict AKI development [6], and it has also outperformed various new markers in predicting bad outcomes [7]. Therefore, FST may be appropriate for risk classification of AKI patients to guide the choice to commence CRRT. Based on the preceding principles, we performed a prospective, double-blind, interventional cohort study to assess the predictive usefulness of FST for CRRT initiation.

## Method

### Trial design and ethics

The double-blind, prospective, interventional cohort study was conducted in the general intensive care unit of the Fourth Hospital of Hebei Medical University, China, between February 2021 and August 2022, collecting patients who met the study's inclusion and exclusion criteria. The study was registered on the China Clinical Trial Registry website: www.chictr.org.cn (ChiCTR1800015734) and authorized by the Fourth Hospital of Hebei Medical University's Ethics Committee. Orally and in writing, the investigator notified the patient or their authorized representative about the experiment.

### Inclusion and exclusion criteria

All AKI patients admitted to the ICU who satisfied the following criteria were assessed for FST. Inclusion criteria: (1) patients admitted to the ICU meeting the AKI diagnostic criteria for Kidney Disease Improving Global Outcomes (KDIGO) guidelines [8]; (2) appropriate blood volume and central venous pressure (CVP) ≥ 6 mmHg; and (3) urine output ≤ 0.5 ml/kg/h for 6 h. Exclusion criteria: (1) indications for emergency CRRT: hyperkalemia, potassium of blood ≥ 6.5 mmol/L; metabolic acidosis, PH ≤ 7.15; acute pulmonary edema due to fluid overload; developed uremia-related complications, such as pericarditis, bleeding, etc.; (2) age < 18 years; (3) during pregnancy or lactation; (4) chronic kidney disease or having received renal replacement therapy 30 days prior to inclusion; (5) patients treated with ECMO; (6) pulmonary embolism; (7) presence of postrenal obstruction factors; (8) the primary disease is irreversible and/or is expected to die within 24 h. If all inclusion criteria were satisfied and there were no exclusion factors, we believed this patient would be eligible for inclusion.

### Furosemide stress test

The urine volume was measured 2 h following administration of a 1 mg/kg intravenous infusion of furosemide (1.5 mg/kg if loop diuretics were used during the previous 7 days) and appropriate fluid resuscitation. FST-responsive if the urine volume was more than 200 mL 2 h after the FST; FST-nonresponsive otherwise [6]. The FST results are recorded and kept secret from the responsible clinician, who selects whether to initiate the CRRT based on laboratory testing and clinical performance other than the FST data. The findings of the FST were concealed from both the subjects and the responsible clinician.

### Study protocol

We established an independent study group to screen patients admitted to the ICU for AKI and collect specimens and perform FST on patients who satisfied the inclusion criteria but did not match the exclusion criteria. The responsible physician and the patient were both blinded to the 2-h urine volume following FST. Patients are not notified of any changes in urine volume following an FST. The study team blinded the physicians by covering the urine bag with an opaque paper bag for 2 h, recording the 2-h urine volume, and then discarding the urine.When the blinding was revealed, the urine volume 2 h after FST was withheld from the critical care record sheet until the patient initiated CRRT or was transferred out of the ICU, at which point the urine volume 2 h after FST was added to the record sheet.

### Biomarkers

We believe that biomarkers may serve as reliable predictors of AKI development and risk stratification. After determining that a patient was eligible, 5 ml of blood was extracted from a vein and spun at 4000 rpm for 10 min. The supernatant was frozen in a refrigerator at − 80 °C, and the neutrophil gelatinase-associated lipocalin (NGAL) concentration was determined using a double-antibody sandwich enzyme linked immunosorbent assay.

### Kidney ultrasound

Kidney ultrasound was then performed, followed by the measurement of the renal resistance index (RRI): locating intrarenal vessels using a convex array probe in two-dimensional ultrasound mode and color Doppler, selecting the interlobe artery or arcuate artery, and measuring renal RRI. RRI = (PSV-EDV)/PSV, where PSV is the peak systolic velocity and EDV is the end-diastolic velocity. The RRI is calculated using the average of three readings [9].

### CRRT parameter setting

The initial CRRT mode is continuous venous-venous hemofiltration (CVVH) using an integrated machine (The Prismaflex^®^ system, Gambro, Sweden or the Aquarius^™^ system, Nikkiso, Japan) filter (AN69) with a prescribed dose set to 25–30 mL/kg/h. The blood flow target was 150–200 mL/min. Local anticoagulant citrate is the first-line anticoagulation method. Patients with citrate anticoagulation contraindications opt for heparin for general anticoagulation, whereas patients with citrate anticoagulation contraindications and a propensity to bleed do not employ an anticoagulation strategy.

### Statistical analysis

The SPSS 26.0 statistical program was utilized for processing and analysis. The Chi-square test or Fisher's exact test is utilized to evaluate categorical data between treatment groups. Continuous variables are presented as means (with standard deviations (SD)) or medians (with inter quartile ranges (IQR)) and compared across groups using the independent t-test for normally distributed data or the Wilcoxon rank sum test for non-normal data. Multivariate analysis was performed using logistic regression. The receiver operating characteristic (ROC) curve was drawn using MEDCALC 15.2.2 software to evaluate the predictive value of CRRT at 2 h after FST. The area under curve (AUC) indicates the predictive value and calculates the sensitivity and specificity of the optimal cut-off value. The *P* < 0.05 was considered as a statistically significant difference.

## Result

### Cohort characteristics and baseline data

**Fig. 1:**
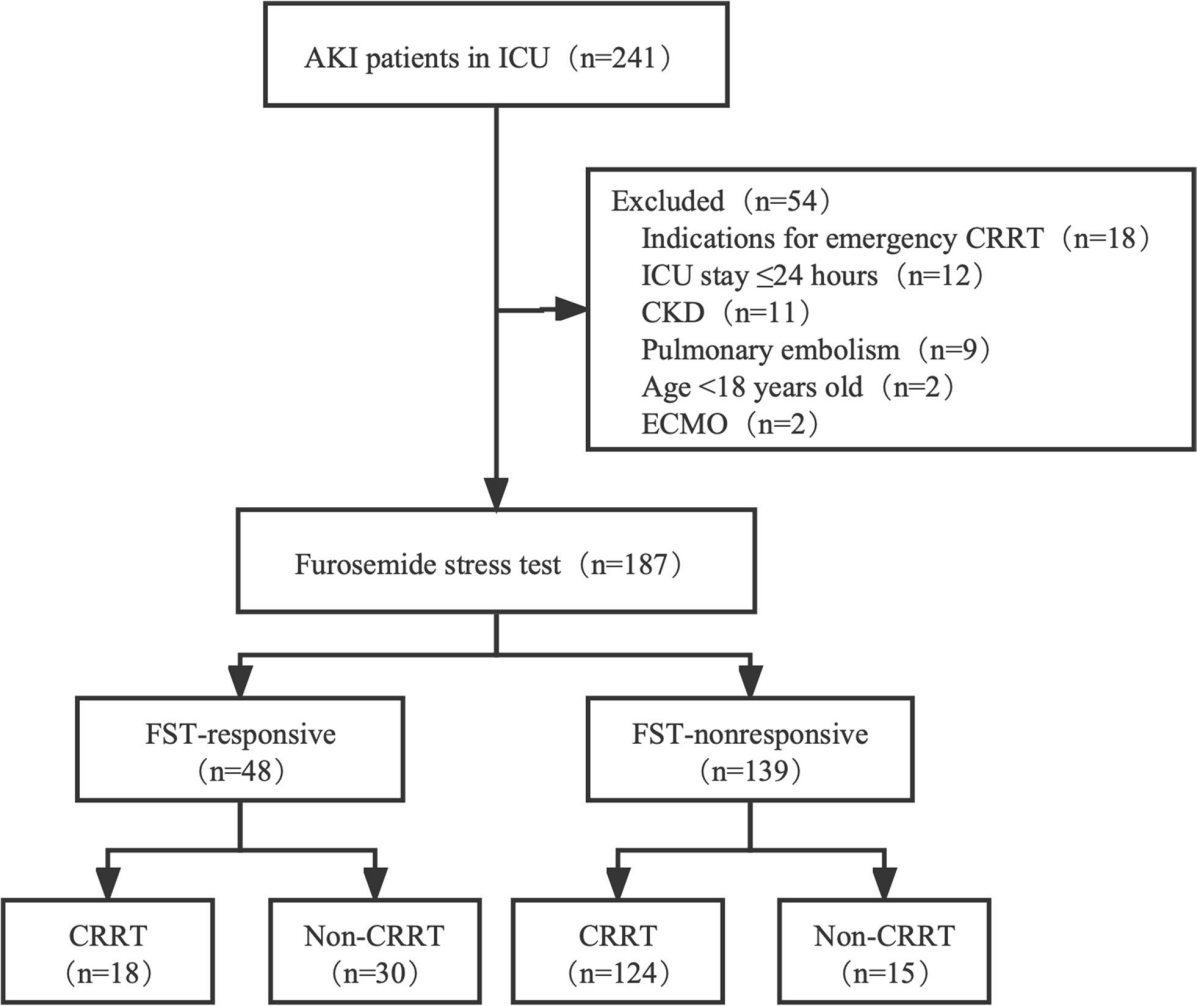
Flowchart of patient allocation. AKI, acute kidney injury; CKD, chronic kidney disease; FST, furosemide stress test; ICU, intensive care unit; CRRT, continuous renal replacement therapy; ECMO, extracorporeal membrane oxygenation

This study examined 241 AKI patients in the ICU; 187 patients got FST; the reasons for not administering FST were as follows: emergency CRRT (18), ICU stay less than 24 h (12), chronic kidney disease (11), pulmonary embolism (9), age less than 18 years (2), and ECMO (2). 18/48 (37.5%) of the FST-responsive patients received CRRT, while 124/139 (89.2%) of the FST-nonresponsive patients received CRRT (Fig. 1).

Baseline data included: general condition, reason for ICU admission, previous medical history, laboratory examination, stage of acute kidney injury, SOFA score, and APACHE II score. The average age of the 187 individuals who had FST was 71 years, and 119 (63.63%) of them were male. 141 (75.4%) patients were admitted to the ICU due to respiratory and circulatory failure. There were 49 (26.20%) cases of AKI in stage 1, 36 (19.25%) in stage 2, and 102 (54.55%) in stage 3. Nearly half of the patients had a previous history of hypertension (47.05%), and 77 (41.18%) had a malignancy. Laboratory examination: white blood cells (15.58 × 10^9^/L, IQR 10–23.18) and procalcitonin (6.85 ng/ml, IQR 1.5–17.03) levels were generally increased, and the mean serum creatinine was 201.4 mmol/L (IQR 100.53–267.4). We tested the mean blood NGAL concentration of 190.66 ng/ml (IQR 138.93–318.77), and ultrasound evaluated the patient with RRI 0.66 (SD 0.08). The proportions of 126 (67.38%) patients with mechanical ventilation and 107 (57.22%) patients with sepsis were relatively high. The mean time from FST to initiation of CRRT was 8.35 (SD 5.75) hours. The SOFA score was 8.58 (SD 0.55), and the APACHE II score was 17.5 (IQR 15–25) (Table 1).

**Table 1:** Baseline information

Variable	All patients (*n* = 187)
General condition	
Age (years), median (IQR)	71 (62.8, 75)
The male, *n* (%)	119 (63.63%)
BMI, mean (SD)	23.91 (0.60)
Temperature (℃), median (IQR)	36.8 (36.5, 37.7)
Heart rate, mean (SD)	107 (4)
Breathing, median (IQR)	20 (15, 21)
Mean arterial pressure (mmHg), mean (SD)	81 (2)
Baseline Scr (μmol/L), median (IQR)	68.85 (52.5, 80.9)
Reason for ICU admission, *n* (%)	
Respiratory failure	86 (45.99%)
Circulatory failure	55 (29.41%)
Neurological diseases	26 (13.9%)
Trauma	8 (4.28%)
Others	12 (6.42%)
Previous medical history, *n* (%)	
Hypertension	88 (47.05%)
Diabetes	37 (19.78%)
Ischemic heart disease	29 (15.51%)
Cerebrovascular disease	53 (28.34%)
Chronic liver disease	4 (2.14%)
Malignancy	77 (41.18%)
Laboratory examination	
WBC (10^9^/L), median (IQR)	15.58 (10, 23.18)
PLT (10^9^/L), median (IQR)	191.5 (107.75, 278.5)
PCT (ng/ml), median (IQR)	6.85 (1.5, 17.03)
Scr (μmol/L), median (IQR)	201.4 (100.53, 267.4)
BUN (mmol/L), median (IQR)	14.55 (8.95, 26.98)
Total bilirubin (μmol/L), median (IQR)	16.1 (10.02, 39.97)
Lactate (mmol/L), median (IQR)	1.85 (1.43, 4.88)
Plasma NGAL (ng/ml), median (IQR)	190.66 (138.93, 318.77)
pH, mean (SD)	7.34 (0.11)
Potassium concentration (mmol/L), median (IQR)	4.38 (4.02, 5.03)
Bicarbonate (mmol/L), median (IQR)	19.8 (15.63, 22.48)
Stage of AKI	
Stage 1, *n* (%)	49 (26.20%)
Stage 2, *n* (%)	36 (19.25%)
Stage 3, *n* (%)	102 (54.55%)
Others	
RRI, mean (SD)	0.66 (0.08)
CVP (mmHg), median (IQR)	9.5 (8, 11)
Mechanical ventilation, n (%)	126 (67.38%)
CRRT, n (%)	142 (75.94%)
Sepsis, n (%)	107 (57.22%)
Time of FST to initiate CRRT (hours), mean (SD)	8.35 (5.75)
SOFA score, mean (SD)	8.58 (0.55)
APACHE II score, median (IQR)	17.5 (15, 25)

BMI, body mass index; ICU, intensive care unit; WBC, white blood cell; PLT, platelet; PCT, procalcitonin; Scr, serum creatinine; BUN, blood urea nitrogen; NGAL, neutrophil gelatinase-associated lipocalin; AKI, acute kidney injury; RRI, renal resistance index; CRRT, continuous renal replacement therapy; SOFA, Sequential Organ Failure Assessment; APACHE II, Acute Physiology and Chronic Health Evaluation II

### Univariate analysis of the CRRT group and the non-CRRT group

The comparison CRRT and non-CRRT groups did not differ significantly in terms of general condition and previous medical history (*P* > 0.05). The platelets were lower in the CRRT group (*P* = 0.04), and the PCT, blood creatinine, blood urea nitrogen, and blood NGAL were all higher than those in the non-CRRT group (*P* < 0.05). There were more acidosis patients in CRRT group (*P* < 0.05). In terms of the stage of acute kidney injury, there were a greater number of stage 2 and stage 3 patients in the CRRT group and a greater number of stage 1 patients in the non-CRRT group. We discovered that urine output after 2 h of FST was significantly lower in the CRRT group than in the non-CRRT group (35 ml, IQR 5–143.75 versus 400 ml, IQR 210–890; *P* = 0.000). The SOFA score and APACHE II score were higher in the CRRT group (*P* < 0.05) (Table 2).

**Table 2:** Univariate analysis of CRRT group and non-CRRT group

Variable	CRRT (n = 142)	non-CRRT (n = 45)	P
General condition			
Age (years), median (IQR)	71 (61.5, 74.5)	68 (62, 76)	0.431
The male, *n* (%)	77 (54.22%)	42 (93.33%)	0.158
BMI, mean (SD)	24.56 (4.82)	25.00 (4.26)	0.760
Temperature (℃), median (IQR)	36.7 (36.4, 37.7)	37.2 (36.5, 37.7)	0.746
Heart rate, mean (SD)	108 (19)	99 (24)	0.151
Breathing, median (IQR)	19 (15, 22)	20 (18, 21)	0.744
MAP (mmHg), mean (SD)	83.24 (10.17)	81.86 (8.68)	0.648
Baseline Scr (μmol/L), median (IQR)	67.4 (52.6, 82.25)	73.6 (50.8, 81.4)	0.867
Reason for ICU admission, *n* (%)			
Respiratory failure	64 (45.07%)	22 (48.89%)	0.654
Circulatory failure	43 (30.28%)	12 (26.67%)	0.643
Neurological diseases	20 (14.08%)	6 (13.33%)	0.899
Trauma	6 (4.23%)	2 (4.44%)	0.950
Others	9 (6.34%)	3 (6.67%)	0.938
Previous medical history, n (%)			
Hypertension	66 (46.48%)	22 (48.88%)	0.778
Diabetes	28 (19.72%)	9 (20.00%)	0.967
Ischemic heart disease	21 (14.79%)	8 (17.77%)	0.629
Cerebrovascular disease	38 (26.76%)	15 (33.33%)	0.394
Chronic liver disease	3 (2.11%)	1 (2.22%)	1.000
Malignancy	60 (42.25%)	17 (37.78%)	0.595
Laboratory examination			
WBC (10^9^/L), mean (SD)	17.87 (10.53)	17.63 (12.73)	0.945
PLT (10^9^/L), median (IQR)	130 (84.25, 229.75)	247.5 (141.25, 367)	0.040
PCT (ng/ml), median (IQR)	7.42 (2.83, 20.81)	2.98 (0.97, 8.69)	0.045
Scr (μmol/L), median (IQR)	267.35 (196.8, 358.52)	100.55 (64.87, 165.2)	0.000
BUN (mmol/L), median (IQR)	18.36 (11.57, 29)	9.2 (6.72, 19.06)	0.009
Total bilirubin (μmol/L), median (IQR)	19.2 (9.1, 46.5)	16.1 (9.75, 34.97)	0.635
Lactate (mmol/L), median (IQR)	1.95 (1.32, 5.47)	1.75 (1.27, 2.22)	0.127
Plasma NGAL (ng/ml), median (IQR)	297.13 (174.79, 485.57)	131.45 (87.36, 200.3)	0.000
pH, mean (SD)	7.32 (0.11)	7.39 (0.06)	0.028
Potassium concentration (mmol/L), median (IQR)	4.46 (4.13, 5.32)	4.3 (3.63, 4.54)	0.074
Bicarbonate (mmol/L), median (IQR)	19.1 (14, 21.65)	20.8 (19.8, 24.2)	0.088
Stage of AKI, *n* (%)			
Stage 1, *n* (%)	16 (11.27%)	33 (73.33%)	0.000
Stage 2, *n* (%)	33 (23.24%)	3 (6.67%)	0.014
Stage 3, *n* (%)	93 (65.49%)	9 (20.00%)	0.000
Others			
FST 2 h urine output (ml), median (IQR)	35 (5, 143.75)	400 (210, 890)	0.000
RRI, mean (SD)	0.68 (0.08)	0.63 (0.08)	0.072
CVP (mmHg), median (IQR)	9 (8, 11)	9 (8, 11)	0.908
Mechanical ventilation, *n* (%)	98 (69.01%)	28 (62.22%)	0.397
Sepsis, *n *(%)	84 (59.15%)	23 (51.11%)	0.342
SOFA score, mean (SD)	10.13 (3.18)	5.86 (2.72)	0.000
APACHE II score, median (IQR)	22.5 (16, 29.5)	16 (15, 18)	0.006

BMI, body mass index; WBC, white blood cell; PLT, platelet; PCT, procalcitonin; Scr, serum creatinine; BUN, blood urea nitrogen; NGAL, neutrophil gelatinase-associated lipocalin; AKI, acute kidney injury; RRI, renal resistance index; CRRT, continuous renal replacement therapy; SOFA, Sequential Organ Failure Assessment; APACHE II, Acute Physiology and Chronic Health Evaluation II

### The RR of CRRT initiation

This study aimed to determine if FST, as an exposure factor, influences doctors' decisions to initiate CRRT. To do this, the relative risk (RR) of the FST not responding to the initiation of CRRT was established. Patients who were FST-nonresponsive were 2.379 times more likely to initiate CRRT than those who were FST-responsive (95% confidence interval [CI], 1.644–3.443, *P* = 0.000).

### Multivariate analysis of the predicted CRRT initiation

As is well known, there are several factors that influence doctors' decisions on the initiation of CRRT. As a result, we included variables that could affect decision-making, omitted confounding variables, and conducted a multivariate analysis. Urinary output 2 h after FST was a predictor of CRRT initiation (*P* = 0.032) (Table 3).

**Table 3:** Multivariate analysis of predicting CRRT initiation

Variable	OR	95% CI	P
Urine output after FST 2 h	0.99	0.98–0.99	0.032
Scr	1.00	0.99–1.02	0.250
PCT	0.99	0.96–1.04	0.885
Plasma NGAL	1.02	0.99–1.04	0.057

FST, furosemide stress testing; Scr, serum creatinine; PCT, procalcitonin; NGAL, neutrophil gelatinase-associated lipocalin

### The ROC of CRRT initiation

**Fig. 2:**
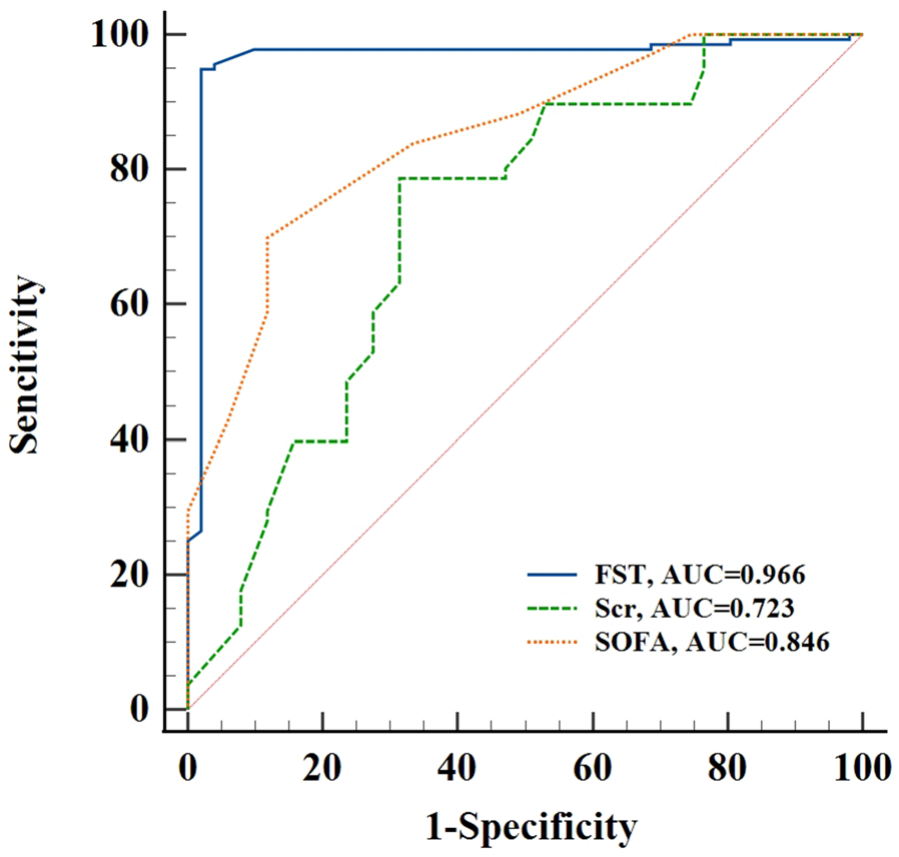
The ROC of urine output after FST 2 h, serum creatinine and SOFA for CRRT initiation. FST, furosemide stress testing; Scr, serum creatinine; SOFA, Sequential Organ Failure Assessment

As we found FST to be an independent and valuable predictor, we investigated a urine output cutoff of 2 h after FST to better direct clinical work. By drawing the receiver operating characteristic (ROC) curve, the area under curve (AUC) was 0.966 (cutoff 156 ml, sensitivity 94.85%, specificity 98.04%, *P* < 0.001). We also analyzed the AUC of serum creatinine and SOFA and discovered that urine production at 2 h after FST had a substantially greater predictive value than the former (Fig. 2) (Table 4).

**Table 4:** The ROC of CRRT initiation

Variable	AUC	SE	95% CI	Cut-off	Sensitivity	Specificity	*P*
Urine output after FST 2 h (ml)	0.966	0.018	0.930–0.987	156	94.85%	98.04%	0.001
Scr (μmol/L)	0.723	0.045	0.653–0.786	115.3	78.68%	68.63%	
SOFA	0.846	0.030	0.786–0.895	8	69.85%	88.24%	

FST, furosemide stress testing; Scr, serum creatinine; SOFA, Sequential Organ Failure Assessment

## Discussion

In recent years, there has been increasing interest in using loop diuretics as trials to assess proximal renal tubular cell injury (e.g., FST) and to predict the progression of AKI. In a follow-up study, urine output at 2 h after FST was superior to the individual urinary biomarker in predicting the progression of stages 1 and 2 AKI to stage 3 AKI [7]. In addition, FST was also used in patients undergoing kidney transplantation to predict the delayed recovery of kidney graft function, defined as receiving RRT within 7 days after kidney transplantation [10]. Therefore, the change in urine volume after FST partly reflects the reserve situation of renal function, especially to avoid the delayed initiation of CRRT [11].

As a result, we designed this prospective interventional cohort study with a double-blind design to prevent FST results from interfering with clinicians initiating CRRT decisions and to ensure that both clinicians and patients were unaware of the FST results. From the results of the study, the feasibility of FST in judging the timing of CRRT initiation is proven. The FST standardization study conducted by Chawla and colleagues showed that the use of 1–1.5 mg/kg of furosemide is safe in patients with AKI [6]. Pharmacologically, furosemide is a chemosynthetic loop diuretic commonly used in clinical practice. The main mechanism of action is to act as by inhibiting the transmembrane domains 11 and 12 of the Na–K–2Cl cotransporter located in the thick wall segment of the renal tubular pulp loop [12]. Inhibiting sodium reabsorption creates a concentration gradient in the renal medulla, which is associated with the main driver of increased water and sodium excretion [13]. We often worry about whether a one-time infusion of furosemide at 1–1.5 mg/kg will burden the kidney and thus further worsen the kidney injury. Indeed, Mariano et al. found that furosemide infusion at 500 mg/24 h was safe with a urine volume greater than 500 ml/24 h and that the diuretic effect was linear with dose. Notably, when the urine volume per 24 h was less than 500 mL, the quantitative-effect relationship vanished and the toxic effect was observed [14]. Similarly, any FST-related safety issues were not observed in the conduct of our study.

Comparing the CRRT group and the non-CRRT group showed a huge difference in urine output at 2 h after FST, which was actually our expected result. The furosemide response has been used to predict renal recovery and stop of RRT in critically ill patients recovering from AKI [15]. Therefore, we designed such a study to validate our idea. Currently, relatively few studies of the FST predict the timing of CRRT initiation; the best known "the FST trial" [16]. The FST was performed on 162 patients with AKI in the ICU, with 118 patients remaining unresponsive. The investigators randomized these 118 people into the early RRT group and the standard RRT group for treatment. Forty-four patients with an FST response were excluded from the randomized cohort, and only 6 of 44 (13.6%) received RRT. Furthermore, patients with non-responders to the FST were highly predictive of requiring RRT, and 45 out of the 60 patients in the standard treatment group ultimately received RRT. FST sorts out severe AKI cases from all AKI cases with high demand for RRT and avoids initiating RRT in low-risk patients. Due to the randomization, the relative risk of initiating CRRT in FST-responsive and FST-nonresponsive patients cannot be compared. Our study, cleverly using a double-blind setting, analyzed the effect of FST as an exposure factor on the outcome of whether to initiate CRRT or not. Ultimately, we found that patients with FST non-responses initiated CRRT 2.379 times more than patients with FST responses. Matsuura et al. [17] reported an OR as high as 11.3, but the 95% CI range was 2.0–65.9. The reasons for this difference may result from the severity of the included cases and the differences in sample sizes. However, our results all suggest that a higher proportion of patients without FST initiate CRRT.

Although FST plays a large role in CRRT initiation and risk stratification in early AKI, some studies have found that it still has appropriate predictive value in AKI stage 3 patients. A retrospective study [18] with a large sample, which included 687 AKI stage 3 patients who underwent FST, observed the predictive value of urine output at 2 h and 6 h after FST for initiating CRRT, with an AUC 0.67 versus 0.71, *P* = 0.02, respectively. The cut-off value at 6 h after the FST was 600 ml, with a sensitivity of 80.9% and a specificity of 50.5%. The proportion of patients with early AKI (stages 1 and 2) and late AKI (stage 3), respectively, was 45.45%, 45%, and 54.55%, and 75.94% of patients received CRRT, indicating that some patients with early AKI received CRRT. Therefore, we plotted the receiver operating characteristic curve to analyze the predictive value and cutoff value of urine volume for CRRT initiation at 2 h after FST. Surprisingly, the area under the curve (AUC) reached 0.966 (cut-off value 156 ml, sensitivity 94.85%, specificity 98.04%, *P* < 0.001).

By identifying potential AKI progression, we can initiate early intervention before life-threatening complications occur. Furthermore, several emerging AKI biomarkers, including tissue inhibitor of metalloproteinases-2 and insulin-like growth factor binding protein-7 (TIMP-2 × IGFBP-7), interleukin-18 (IL-18), and plasma NGAL, have been shown to predict the progression of AKI [19–21]. In particular, TIMP-2 × IGFBP-7 was confirmed for early AKI risk stratification in critically ill patients in multicenter studies and have been approved by the US FDA for clinical use [22, 23]. These cell cycle arrest biomarkers are expected to facilitate the early detection of patients at risk for AKI in a variety of clinical settings. Therefore, plasma NGAL was also detected in our study, and although there were statistically significant differences between the CRRT and non-CRRT groups (*P* < 0.05), plasma NGAL did not show superiority when we performed a multivariate analysis affecting the timing of CRRT initiation.

This study has some limitations: first, this is a single-center prospective cohort study with a double-blind design that was limited by a small single-center sample size and an offset in sample characteristics (for example, 41.18% of participants had a malignancy). As a result, the study's findings must be validated in a larger sample size and multicenter study. Second, although we designed the RRI, the two groups did not show significant differences. There are also studies showing a limited predictive value of RRI in AKI progression [24, 25]. Limited by conventional ultrasound resolution, which does not show very well for changes in renal cortical microcirculation, perhaps we should further evaluate renal perfusion in using microbubble contrast ultrasound imaging [26]. From a pathophysiological point of view, the renal blood flow should change [27].

## Conclusion

This study shows that FST is a safe and practical way to figure out when CRRT should be started in critically ill AKI patients.

## Data Availability

All data generated and/or analyzed during this study are included in this published article.

## Abbreviations

CRRT: Continuous renal replacement therapy
AKI: Acute kidney injury
FST: Furosemide stress test
ICU: Intensive care unit
KDIGO: Kidney Disease Improving Global Outcomes
CVP: Central venous pressure
NGAL: Neutrophil gelatinase-associated lipocalin
RRI: Renal resistive index
PSV: Peak systolic velocity
EDV: End diastolic velocity
CVVH: Continuous venous-venous hemofiltration
SD: Standard deviations
IQR: Inter quartile ranges
ROC: Receiver operating characteristic
AUC: Area under curve
SOFA: Sequential Organ Failure Assessment
APACHE II: Acute Physiology and Chronic Health Evaluation II
ECMO: Extracorporeal membrane oxygenation
CKD: Chronic kidney disease
BMI: Body mass index
WBC: White blood cell
PLT: Platelet
PCT: Procalcitonin
Scr: Serum creatinine
BUN: Blood urea nitrogen
TIMP-2: Tissue inhibitor of metalloproteinases-2
IGFBP-7: Insulin-like growth factor binding protein-7
IL-18: Interleukin-18
